# Frequent Occurrence and Metabolic Versatility of *Marinifilaceae* Bacteria as Key Players in Organic Matter Mineralization in Global Deep Seas

**DOI:** 10.1128/msystems.00864-22

**Published:** 2022-11-07

**Authors:** Jianyang Li, Chunming Dong, Qiliang Lai, Guangyi Wang, Zongze Shao

**Affiliations:** a Key Laboratory of Marine Genetic Resources, Third Institute of Oceanography, Ministry of Natural Resources of PR China, Xiamen, People’s Republic of China; b State Key Laboratory Breeding Base of Marine Genetic Resources, Xiamen, People’s Republic of China; c Key Laboratory of Marine Genetic Resources of Fujian Province, Xiamen, People’s Republic of China; d School of Environmental Science and Engineering, Tianjin University, Tianjin, People’s Republic of China; e Southern Marine Science and Engineering Guangdong Laboratory (Zhuhai), Zhuhai, People’s Republic of China; Oregon State University

**Keywords:** deep sea, *Marinifilaceae*, biodiversity, organic matter mineralization, lignin oxidation, nitrogen fixation

## Abstract

Transfer of animal and plant detritus of both terrestrial and marine origins to the deep sea occurs on a global scale. Microorganisms play an important role in mineralizing them therein, but these are yet to be identified *in situ*. To observe key bacteria involved, we conducted long-term *in situ* incubation and found that members of the family *Marinifilaceae* (MF) occurred as some of the most predominant bacteria thriving on the new inputs of plant and animal biomasses in the deep sea in both marginal and oceanic areas. This taxon is diverse and ubiquitous in marine environments. A total of 11 MAGs belonging to MF were retrieved from metagenomic data and diverged into four subgroups in the phylogenomic tree. Based on metagenomic and metatranscriptomic analyses, we described the metabolic features and *in situ* metabolizing activities of different subgroups. The MF-2 subgroup, which dominates plant detritus-enriched cultures, specializes in polysaccharide degradation and lignin oxidation and has high transcriptional activities of related genes *in situ*. Intriguingly, members of this subgroup encode a nitrogen fixation pathway to compensate for the shortage of nitrogen sources inside the plant detritus. In contrast, other subgroups dominating the animal tissue-supported microbiomes are distinguished from MF-2 with regard to carbon and nitrogen metabolism and exhibit high transcriptional activity for proteolysis *in situ*. Despite these metabolic divergences of MF lineages, they show high *in situ* transcriptional activities for organic fermentation and anaerobic respiration (reductions of metal and/or dimethyl sulfoxide). These results highlight the role of previously unrecognized *Marinifilaceae* bacteria in organic matter mineralization in marine environments by coupling carbon and nitrogen cycling with metal and sulfur.

**IMPORTANCE** Microbial mineralization of organic matter has a significant impact on the global biogeochemical cycle. This report confirms the role of *Marinifilaceae* in organic degradation in the oceans, with a contribution to ocean carbon cycling that has previously been underestimated. It was the dominant taxon thriving on plant and animal biomasses in our *in situ* incubator, as well as in whale falls and wood falls. At least 9 subgroups were revealed, and they were widely distributed in oceans globally but predominant in organic-matter-rich environments, with an average relative abundance of 8.3%. Different subgroups display a preference for the degradation of different macromolecules (polysaccharides, lignin, and protein) and adapt to their environments via special metabolic mechanisms.

## INTRODUCTION

Deep-sea environments host abundant microbial communities which play a major role in the global material and element biogeochemical cycles (1). The principal source of carbon and energy for the dark sea biota is primary production from the upper layer of the ocean and the resulting deposition of particulate organic matter (POM), dissolved organic matter (DOM), and refractory dissolved organic matter (rDOM) via multiple pumps (2, 3). The organic matter (OM) input into the deep sea, including POM, DOM, and rDOM, critically contributes to the establishment of habitats for deep-sea microbial communities (4).

Heterotrophic microorganisms play a keystone role in the mineralization of OM in the deep sea, particularly the dissolution of POM to DOM and subsequent absorption of cleavage products, both of which are considered rate-limiting steps of deep-sea microbial metabolism (5). OM remineralization in the oceans occurs mainly via marine heterotrophic bacteria, either aerobically or anaerobically. DOM, which usually consists of diverse low-molecular-weight compounds, is largely subjected to aerobic microbial degradation, while POM, such as proteins, polysaccharides, and lipids inside insoluble particles, tends to go through the anaerobic process even in oxic seawater (6). The particle-associated microorganisms in the deep sea have been examined by filter fractionation techniques and sediment traps (5, 7–13). The sediment trap study indicated that the piezophile-like *Gammaproteobacteria* and *Epsilonproteobacteria* play a central role in the mineralization of sinking POM in the deep sea (12). Another report showed that particle-associated microorganisms, such as *Sphingomonadales*, *Rhodobacterales*, and *Alteromonadales*, play a primary role in the cleavage of OM by releasing their extracellular enzymes into the particles in the bathypelagic realm (13).

In comparison, little was known about the microbial-mediated mineralization of large and fast-sinking particles, such as oceanic or terrestrial animal and plant tissues like well-known ecosystems created by wood fall, kelp fall, and whale fall (14–18). The breakdown of animal and plant input into the deep sea occurs repeatedly on a global scale every year; these events and their locations are regarded as “hot times” and “hot spots,” respectively. The large and fast-sinking particles may escape disaggregation, dissolution, and solubilization en route to the deep sea (19) and impact the microbial communities in the deep sea (20, 21). Around 500 Tg C from terrestrial OM is imported into the ocean annually, and ca.10% is buried in marine sediments (22, 23). Although this amount is less than POM and DOM both derived from primary production in the sunlit surface waters of the ocean, the impact of terrestrial OM input into the deep sea cannot be ignored (24), as well as that from kelp fall and whale fall. Some studies have estimated that a single storm event could transport up to 1.8 to 4 Tg of driftwood carbon to the ocean and that a single large whale carcass could provide a flux equivalent to 2,000 years of background POM flux, or 30 to 40% of the annual carbon flux of a swarm of sinking swimming crabs to the deep ocean floor (25). Recently, a report estimated that 4.7 million m^3^ of large wood could flow into the oceans each year (26). These estimates clearly demonstrate the importance of large organic falls to the ecology of deep-sea ecosystems. However, wood has been unheeded in the quantification of organic carbon burial on continental margins (27).

The degradation of OM derived from such input remains is a temporally dynamic process that entails a succession of specialized communities with distinct lifestyles and metabolic potentials in the deep sea (28). Localized, high organic concentrations may deplete oxygen, which in turn attracts anaerobic microbial communities thriving on the organic falls (29, 30). These resulting habitats are described as chemosynthetic ecosystems because of the sulfide production that finally inducing chemosynthetic communities (18, 31). However, the mineralization mediated by microbes remains less explored in the deep sea, which hampers our ability to evaluate the fate and impacts of those bulky remains.

We hypothesized that the microorganisms thriving on remains sunk into the deep sea may be different from the bacteria observed in the deep water column sampled with CTD cassettes, filter fractionation techniques, and sediment traps, because large pieces rich in OM do not constantly sink through the water column and settle to the seabed at every location. To identify the bacterial assemblage involved in the *in situ* transformation and degradation of the fast-sinking POM in the deep sea, we performed deep-sea *in situ* incubation amended with various natural animal and plant tissues in the seabed of the Indian Ocean (at a depth of 4,434 m), Pacific Ocean (1,622 m), and South China Sea (3,758 m) (32). Four to 12 months of deep-sea *in situ* incubations resulted in anaerobic environments, containing large numbers of anaerobes and facultative anaerobes, such as sulfur-oxidizing bacteria (SOB) of *Arcobacteraceae* and *Sulfurovaceae*, and sulfate-reducing bacteria (SRB) of *Desulfobulbaceae* and *Desulfobacteraceae*. In particular, bacteria of the family *Marinifilaceae* (MF) were the predominant members of both animal and plant tissue enrichments. Whether and how this bacterial group plays a role in the depolymerization, degradation, and transformation of macromolecular OM remain uncertain. To date, only a few bacteria of this family have been isolated (33), such as Labilibaculum manganireducens (34), Ancylomarina subtilis (35), and Marinifilum albidiflavum (36), but these are distantly related to the ones in our *in situ* cultures. In this study, we analyzed the genetic diversity, global distribution, environmental adaptation, and metabolism of MF via phylogenetic, metagenomic, and metatranscriptomic analyses. This knowledge will facilitate our understanding of the role of these organisms in biogeochemical cycles in oceanic interiors.

## RESULTS AND DISCUSSION

### *In situ* incubation of deep-sea OM-mineralizing bacteria.

*In situ* incubation was conducted with a deep-sea *in situ* microbial incubator (DIMI) amended with wood chips, wheat bran, fish scales, fish tissue, and fish oil (including docosahexaenoic acid [DHA] and eicosapentaenoic acid [EPA]) on a flat-topped seamount in the west Pacific Ocean (1,622 m water depth and 2.44°C), on the seafloor in the South China Sea (3,758 m water depth and 2.39°C), and on the deep-sea basin beside the southwest Indian Ridge in the Indian Ocean (4,434 m water depth and 2.35°C) for 348, 375, and 117 days, respectively (32). After that, the DIMI was autorecovered to the research vessel and the *in situ* enrichments were subjected to community composition analysis. As a result, MF bacteria were found to be prevalent and dominant in the enriched assemblages (32). Subsequently, the diversity and global distribution pattern of MF were analyzed based on our *in situ* data and public NCBI and IMNGS (Integrated Microbial Next Generation Sequencing) databases (see Tables S1 and S2 at https://doi.org/10.5281/zenodo.7050448). We obtained 11 metagenome-assembled genomes (MAGs) belonging to MF bacteria from our metagenomic data and public data from wood fall and analyzed the *in situ* metabolisms based on metatranscriptomic data.

### Diversity and wide distribution of MF bacteria in the global ocean.

**FIG 1:**
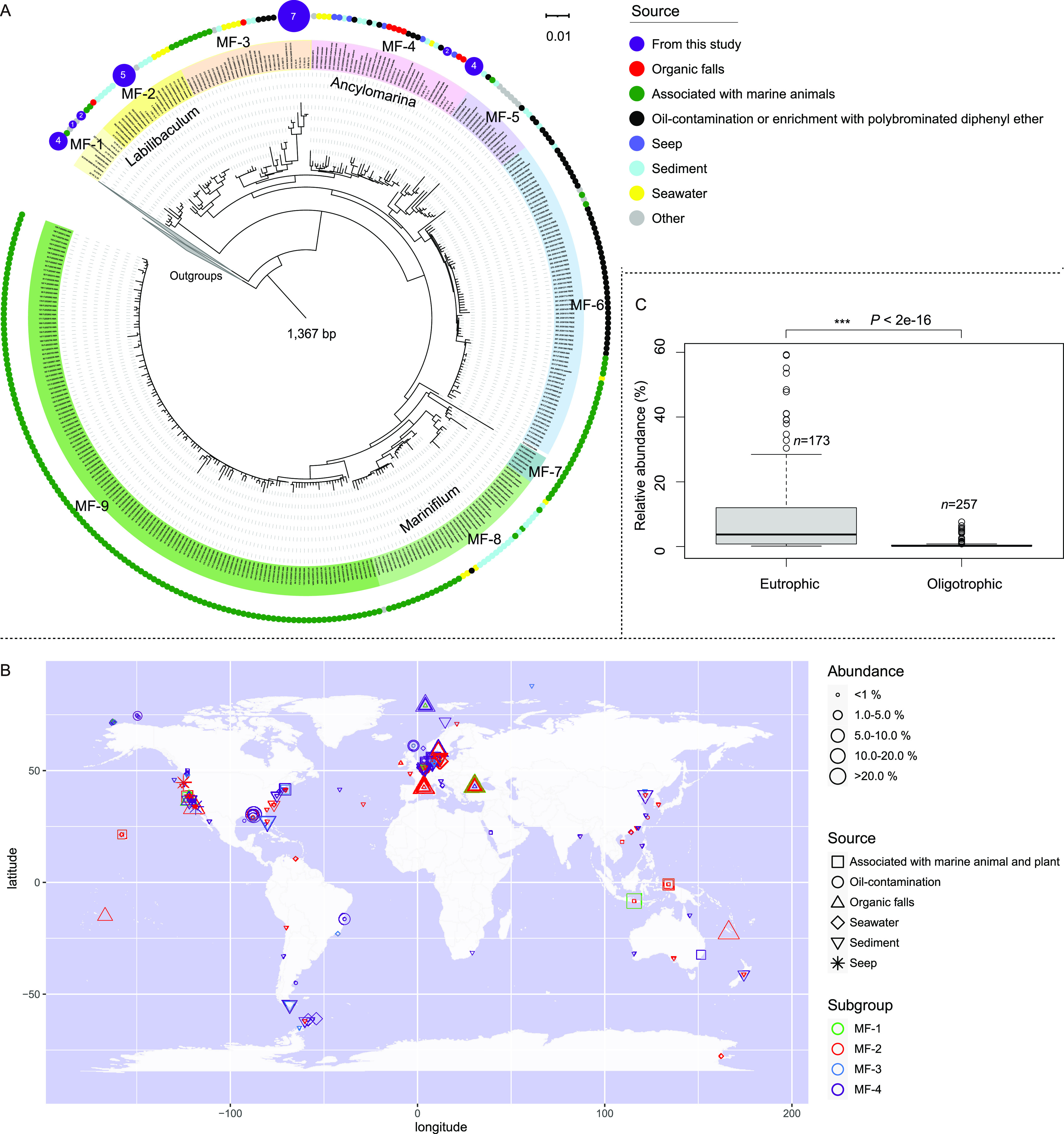
Phylogenetic relationships and global distribution pattern of MF-1 to MF-4 based on 16S rRNA sequence metadata. (A) Phylogenetic tree based on the near-full-length 16S rRNA (1,367 bp) gene sequences within the MF clade. There are at least 9 subgroups within the MF clade. MF-2, MF-4, and MF-8 are closely related to the cultured bacteria of the genera *Labilibaculum*, *Ancylomarina*, and *Marinifilum*, respectively, while members of other subgroups remain uncultivated, and these subgroups might represent novel genera of this family. The families *Prolixibacteraceae* and *Marinilabiliaceae* were used as the outgroups. (B) Global distribution of MF-1 to MF-4. Bacteria of four subgroups are widely distributed in marine environments, including seawater column, marine sediment, marine animal and plant surface or animal intestine, and other OM-rich marine habitats, such as whale falls, wood falls, seagrass detritus, cold seeps, and petroleum-contaminated sediment, but they are not found in terrestrial and freshwater environments. There metadata were all retrieved from environmental samples. (C) Variation analysis of the relative abundance of MF from the eutrophic and oligotrophic environments. The average abundance of these MF members was significantly higher in eutrophic environments than in oligotrophic sediment and seawater. Welch’s *t* test was used to estimate the significance among the samples. ***, *P* ≤ 0.001.

The neighbor-joining trees based on 16S rRNA gene sequences showed that the clones belonging to MF were closely related to the genera *Labilibaculum* and *Ancylomarina* (see Fig. S1 at https://doi.org/10.5281/zenodo.7050448) and that MF contained at least 9 subgroups (tentative genus level), named MF-1 to MF-9 (Fig. 1A). Among the subgroups, MF-2, MF-4, and MF-8 were closely related to cultured bacteria of the genera *Labilibaculum*, *Ancylomarina*, and *Marinifilum*, respectively, while other subgroups have no pure cultures to match and might represent novel genera of this family. In particular, the MF bacteria detected in the five *in situ* enrichments were affiliated with the MF-1, MF-2, MF-3, and MF-4 subgroups.

The global distribution pattern of MF-1, MF-2, MF-3, and MF-4 showed that they are widely distributed in marine environments, including seawater columns, marine sediments, marine animal and plant surfaces or animal intestines, and OM-rich marine habitats, such as whale falls, wood falls, seagrass detritus, cold seeps, and petroleum-contaminated sediment, but they are not found in terrestrial and freshwater environments (Fig. 1B; also, see Tables S1 and S2 at https://doi.org/10.5281/zenodo.7050448). Moreover, based on the metadata (see Table S1 at https://doi.org/10.5281/zenodo.7050448), we found that the average abundance of the four subgroups in OM-rich environments, e.g., wood falls, whale falls, oil pollution, and enrichments with seaweed or polysaccharide, was 8.3%, which was significantly (*P < *0.001) higher than that in oligotrophic sediment and seawater (Fig. 1C). Recently, Yadav et al. reported that MF members were present throughout the water column from 5 m to 2,000 m in the Black Sea, with a maximum cell abundance of ~1 × 10^8^ copies/mL (37). In the communities of wood fall (38), whale fall (15), and the sediment contaminated with oil (39), the relative abundance of MF bacteria reached 28%, 11%, and 41%, respectively. These results implied that MF bacteria are opportunistic bacteria that may exist as rare and ubiquitous species during periods of famine, waiting for the feast of sinking bulky POM in the deep sea (4) and that they may play an important role in the oceans.

### Preference of MF subgroups for different organic substrates.

**FIG 2:**
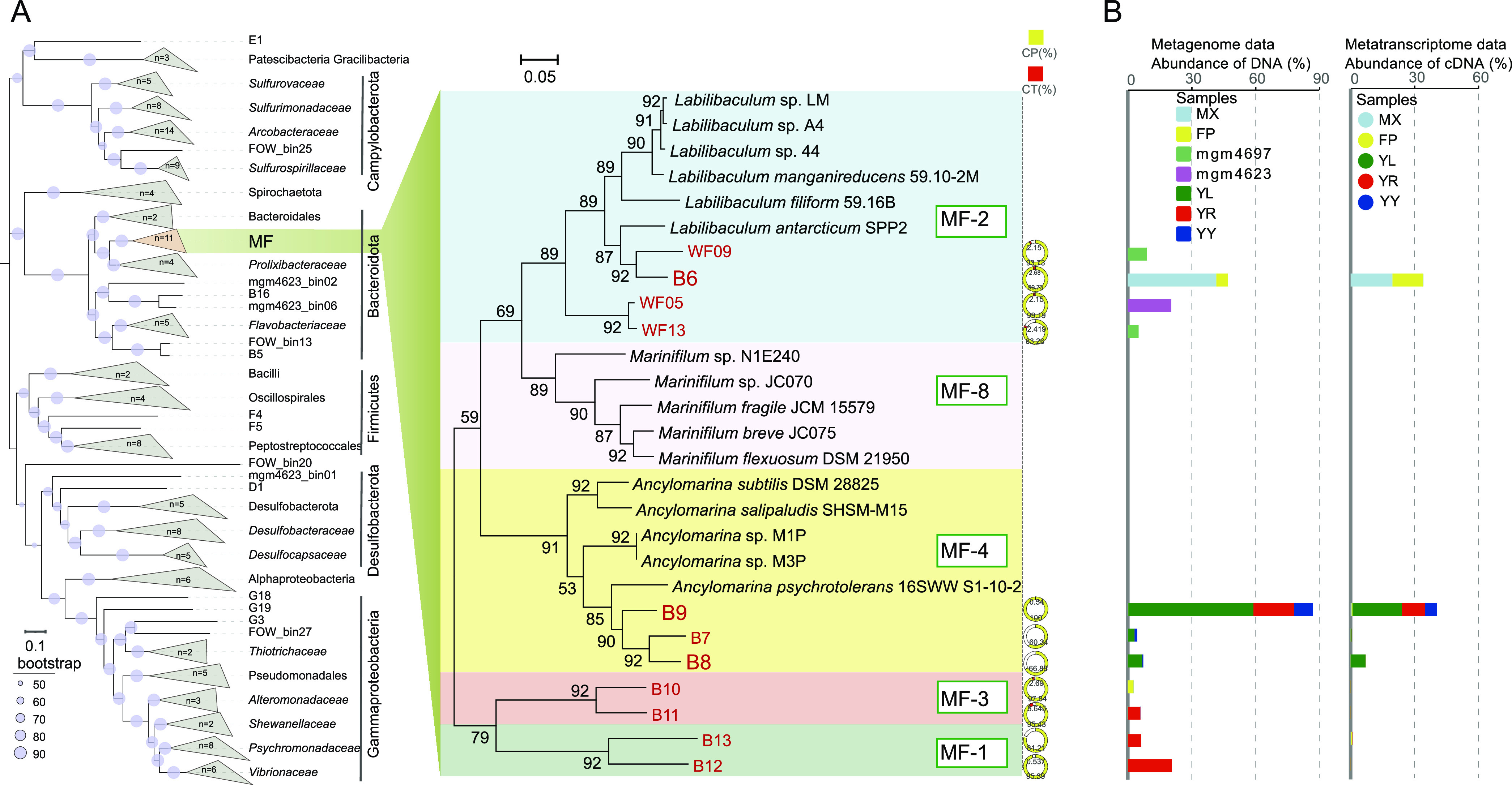
Phylogenomic tree and abundance profiles of the taxon MF in the OM-enriched assemblages. (A) Phylogenetic trees for all the MAGs obtained in this study (left) and for MF lineage including 11 MAGs and 16 genomes from pure cultures (right). The numbers in each clade represent the number of MAGs from this study. Phylogenetic trees were constructed by using concatenated marker genes. WF13, WF05, and WF09 were retrieved from wood falls (18, 38), B6 to B13 are from our *in situ* enrichments in this study, and others are isolated strains. CP, completeness of MAGs; CT, contamination of MAGs. (B) Abundance profiles of MF members in the OM-enriched assemblages with wood chips, wheat bran, fish scales, fish tissue, and fish oil *in situ* in the deep sea based on metagenomic (left) and transcriptomic (right) data sets. MX, wood chips; FP, wheat bran; YL, fish scales; YR, fish tissue; YY, fish oil (DHA and EPA). mgm4697 and mgm4623 are the public wood falls.

A total of 11 distinct MAGs (bin B6 to bin B13, WF05, WF09, and WF13) belonging to MF were obtained from the five enrichments in this study and two wood fall samples (18, 38) (Table 1; also, see Table S3 at https://doi.org/10.5281/zenodo.7050448). These MAGs together with the genomes of 16 cultured strains were clustered into five subgroups (MF-1 to MF-4 and MF-8) in the phylogenomic tree (Fig. 2A). Each subgroup might represent a different genus. Among them, MF-2, MF-4, and MF-8 were related to the genera *Labilibaculum*, *Ancylomarina*, and *Marinifilum*, respectively, while both MF-1 and MF-3 contained no pure culture and formed a separate clade against MF-2 and others. Therefore, they might represent two novel genera. These 11 MAGs represent 10 different novel species based on the average nucleotide identity (ANI) value (WF13 and WF05 belonged to the same species according to their high ANI value) (see Table S4 at https://doi.org/10.5281/zenodo.7050448).

**TABLE 1:** Genomic features and metabolic potential of MF MAGs^*a*^

Feature	MF-2			MF-4			MF-3		MF-1	
	B6	WF09	WF05	B7	B8	B9	B10	B11	B12	B13
Total length (bp)	6,404,378	5,810,170	4,508,729	4,058,876	4,530,168	4,259,741	3,848,611	4,421,923	3,681,417	3,982,496
No. of scaffolds	71	33	181	300	386	74	225	151	122	282
Completeness (%)	99.73	93.73	99.19	60.34	66.88	100	97.84	95.43	95.39	81.22
Contamination (%)	2.688	2.150	2.150	0	0	0.537	2.688	5.645	0.537	0
GC content (%)	35.2	34.5	36.7	34.3	34.6	34.5	31.4	31.2	31.3	29.3
*N*_50_ (bp)	139,878	10,854	26,354	22,685	20,944	78,072	22,697	44,719	40,423	22,542
No. of predicted genes	4,836	4,699	3,498	3,443	3,662	3,468	2,824	3,250	2,600	2,910
EggNOG (%)	4,283 (88.6)	3,959 (84.3)	3,135 (89.6)	2,955 (85.8)	3,111 (85.0)	3,031 (87.4)	2,447 (86.7)	2,816 (86.6)	2,300 (88.5)	2,492 (85.6)
Pfam (%)	3,740 (77.3)	3,439 (73.2)	2,667 (76.2)	2,565 (74.5)	2,672 (73.0)	2,653 (76.5)	2,112 (74.8)	2,422 (74.5)	2,001 (77.0)	2,184 (75.1)
Lignin	+	+	+	−	−	+	−	−	−	−
Xylan	+	+	+	−	−	−	−	−	−	−
Cellulose	+	+	+	−	−	−	−	−	−	−
l-Arabinose	+	+	+	−	−	−	−	−	−	−
d-Xylose	+	+	+	+	+	−	−	−	−	−
d-Fructose	+	+	+	+	+	+	+	+	+	+
Metal reductase	+	+	−	−	−	+	+	+	+	+
Thiosulfate reductase	+	+	+	+	+	+	−	−	−	−
Dissimilatory nitrate reduction	+	+	−	+	−	+	−	−	−	−
H_2_ production	+	+	+	+	+	+	−	−	−	−
Nitrogen fixation	+	+	+	−	−	−	−	−	−	−
Hydroxylamine reductase	+	+	+	+	+	+	+	−	−	+
Assimilative nitrate reduction	+	−	−	−	−	−	−	−	−	−
Cobalamin synthesis	+	+	+	−^*b*^	−^*b*^	−^*b*^	−^*b*^	−^*b*^	−^*b*^	−^*b*^

a +, positive; −, negative.

b The cobalamin synthesis pathway is incomplete.

These MF MAGs were predominant in those five microbial assemblages, exhibiting 8.4 to 68.5% genomic DNA abundance (see Table S5 at https://doi.org/10.5281/zenodo.7050448). In particular, the relative abundance of MAG B6 and B9 was as high as 42% and 59% in wood chip and fish scale enrichments, respectively (Fig. 2B; also, see Table S5 at https://doi.org/10.5281/zenodo.7050448). Similarly, WF05, WF09, and WF13 had 5 to 21% relative abundances in the wood fall assemblages (18, 38). Moreover, the relative cDNA abundance of the MAG B6 and B9 was 14.3 to 19.7% and 10.9 to 23.2% in the enrichments with polysaccharide-rich and protein-rich substrates, respectively (Fig. 2B; also, see Table S5 at https://doi.org/10.5281/zenodo.7050448). MAG B9 also accounted for 7.1% of the total transcripts in the enrichment with fish oil, and MAG B8 accounted for 7.0% in the enrichment with fish scales (Fig. 2B; also, see Table S5 at https://doi.org/10.5281/zenodo.7050448). However, different subgroups showed a preference for organic substrates. B6, WF05, WF09, and WF13, all affiliated with MF-2, were enriched only with the plant detritus (wood chips and wheat bran), while others were enriched with the animal tissue (fish tissue and fish scales) and/or fish oil (Fig. 2B). This indicated the existence of different metabolic mechanisms in different subgroups.

The studies described below investigated the metabolic mechanisms of subgroups MF-1 to MF-4 to identify their adaptation to deep-sea environments and their ecological roles *in situ* based on the annotation of the draft genomes and metatranscriptomic data.

### MF-2 bacteria involved in the hydrolysis of polysaccharides.

MF-2 bacteria could encode 527 to 1,041 carbohydrate-active enzymes, which was more than that in the other three subgroups (MF-1, -3, and -4) (see Table S6 at https://doi.org/10.5281/zenodo.7050448). Moreover, the genes encoding glycoside hydrolases (GHs) were significantly (*P < *0.001) enriched in MF-2 bacteria compared to the other three subgroups (see Fig. S2 at https://doi.org/10.5281/zenodo.7050448), which implied that MF-2 possesses a high potential for polysaccharide hydrolysis and substrate range. No GH family associated with cellulose or xylan hydrolysis was found in bacteria of MF-1, -3, and -4, but many were found in MF-2 bacteria, e.g., GH43, GH5, GH26, GH9, GH10, and GH51 (see Table S6 at https://doi.org/10.5281/zenodo.7050448).

MAG B6, which was dominant in the consortia enriched with wood chips and wheat bran, harbored 263 GH genes, more than half of which were predicted to contain signal peptide sequences (see Table S7 at https://doi.org/10.5281/zenodo.7050448). These GH genes were assigned to 60 GH families and formed at least 32 polysaccharide utilization loci (PULs) (see Fig. S3 and S4 at https://doi.org/10.5281/zenodo.7050448), which was more than in *Polaribacter* MAGs (fewer than 10 PULs), which thrives on spring algal blooms (17). These PULs in MAG B6 were mostly involved in the hydrolysis of hemicellulose, pectin, starch, cellulose, polysaccharides containing *N*-acetyl groups (such as peptidoglycan and chitin), and various oligosaccharides (see Fig. S4 at https://doi.org/10.5281/zenodo.7050448). Moreover, like other marine *Bacteroidetes* bacteria present in algal blooms (17, 40, 41), MAG B6 could encode at least 34 pairs of SusCD-like complexes (see Fig. S4 at https://doi.org/10.5281/zenodo.7050448), which were found to be responsible for binding and transporting oligosaccharides into cells (42). A study on wood fall showed that there were also many SusCD-like genes present in the metagenome data (18).

**FIG 3:**
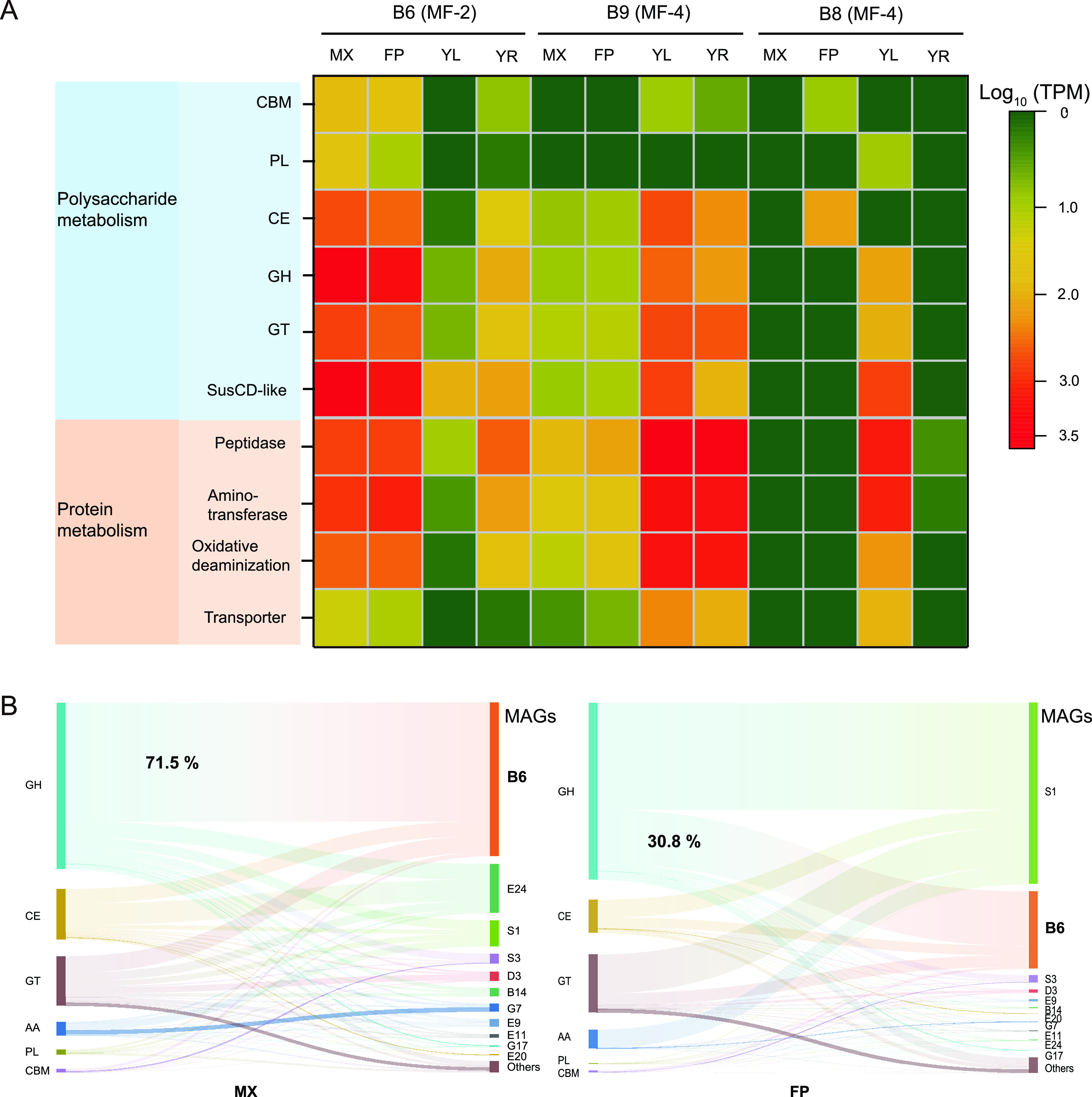
Transcriptional profiles of metabolic pathways involving polysaccharide and protein degradation. (A) Metabolic transcriptional profiles associated with polysaccharide and protein degradation for the dominant MF members B6, B8, and B9 in four POM-enriched assemblages with wood chips, wheat bran, fish scales, and fish tissue. (B) Transcriptional distribution patterns of gene sets related to polysaccharide degradation in different MAGs in MX- and FP-enriched consortia. MX, wood chips; FP, wheat bran; YL, fish scales; YR, fish tissue. GH, glycoside hydrolase; CE, carbohydrate esterase; GT, glycosyltransferase; CBM, carbohydrate-binding module; PL, polysaccharide lyase; AA, auxiliary activity.

**FIG 4:**
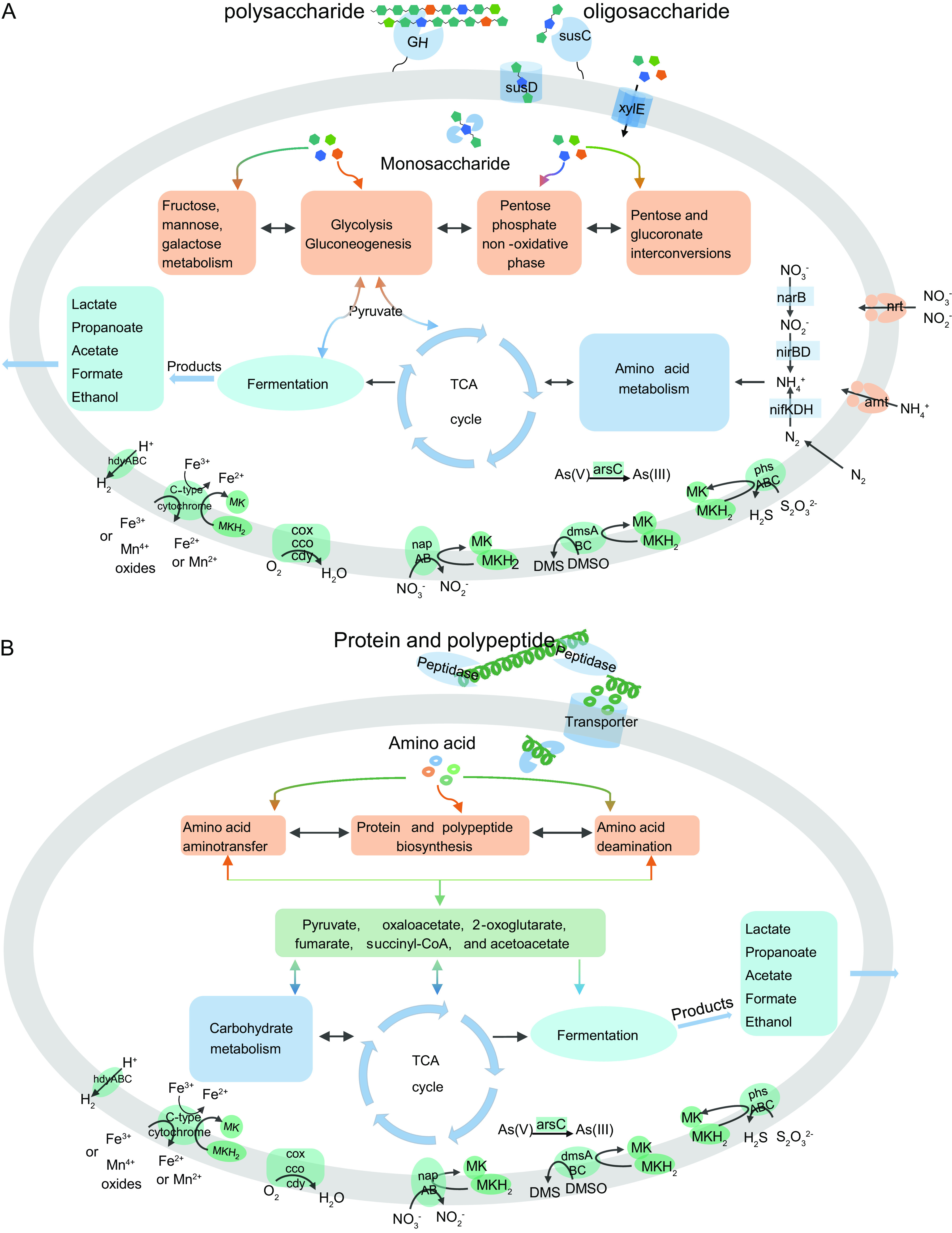
Metabolism of MF B6 and B9. (A) *In situ* depolymerization, degradation, and transformation of extracellular polysaccharides via MF B6 coupled with various respiration, fermentation, and other elemental cycling processes in the deep sea. (B) *In situ* depolymerization, degradation, and transformation of extracellular protein or polypeptide via MF B9 coupled with various respiration, fermentation, and other elemental cycling processes in the deep sea. MF B6 and B9 were the dominant species in plant detritus and animal tissue enrichments, respectively. They are the keystones in the degradation of polysaccharide POM and protein POM *in situ* in the deep sea. Both nitrogen fixation and nitrate assimilation pathways were present in MF B6 but not in MF B9. CoA, coenzyme A; TCA, tricarboxylic acid.

Consistent with the genomic features for saccharide metabolism, all the genes in MAG B6 involved in the hydrolysis of polysaccharides, in addition to oligosaccharide and monosaccharide metabolism, were actively transcribed in the wood chip and wheat bran consortia (Fig. 3A; also, see Tables S7 and S8 at https://doi.org/10.5281/zenodo.7050448). In particular, 71.5% and 30.8% of transcripts assigned to GH genes in the wood chip and wheat bran consortia, respectively, were generated from MAG B6 (Fig. 3B). The genes involved in the hydrolysis of hemicellulose, pectin, starch, *N*-acetyl-containing polysaccharides, and oligosaccharides occupied >89% of the total GH transcripts in MAG B6 (see Fig. S5 at https://doi.org/10.5281/zenodo.7050448). The polysaccharide metabolism via MAG B6 is shown in Fig. 4A. These results indicated that MAG B6 contributed significantly to the degradation of plant detritus *in situ* in the deep sea.

Among the GH genes in MAG B6, one (gene ID 27_12) exhibited the highest and second highest transcriptional activity in the wheat bran and wood chip consortia, respectively (see Table S7 at https://doi.org/10.5281/zenodo.7050448). This gene is closely related to the gene *xyn10b* (PDB ID 2W5F) in Clostridium thermocellum (43) and encodes a carbohydrate-binding module (CBM) domain and an endo-1,4-beta-xylanase catalytic domain affiliated with the GH10 family (see Fig. S6 at https://doi.org/10.5281/zenodo.7050448). This result indicated this xylanase played a major role in the degradation of plant detritus *in situ*.

### Potential for lignin oxidation by subgroup MF-2.

Since lignin is one of the main components in wood chips, we also analyzed whether MF B6 has the potential to oxidize lignin. The genome data showed that one manganese superoxide dismutase (MnSOD) gene and one catalase-peroxidase (KatG) gene were detected in MAG B6, both of which have been confirmed to oxidize lignin and/or lignin derivatives (44, 45). MnSOD encoded by MAG B6 shared a high amino acid similarity of 64.0% with two MnSODs from *Sphingobacterium* sp. strain T2 and also contained structurally and functionally important residues (see Fig. S7 at https://doi.org/10.5281/zenodo.7050448). *Sphingobacterium* sp. strain T2 could produce hydroxyl radical from hydrogen peroxide via its MnSODs, and then the hydroxyl radical oxidizes the lignin via C_α_-C_β_ and aryl-C_α_ cleavage and demethylation (44, 46).

**FIG 5:**
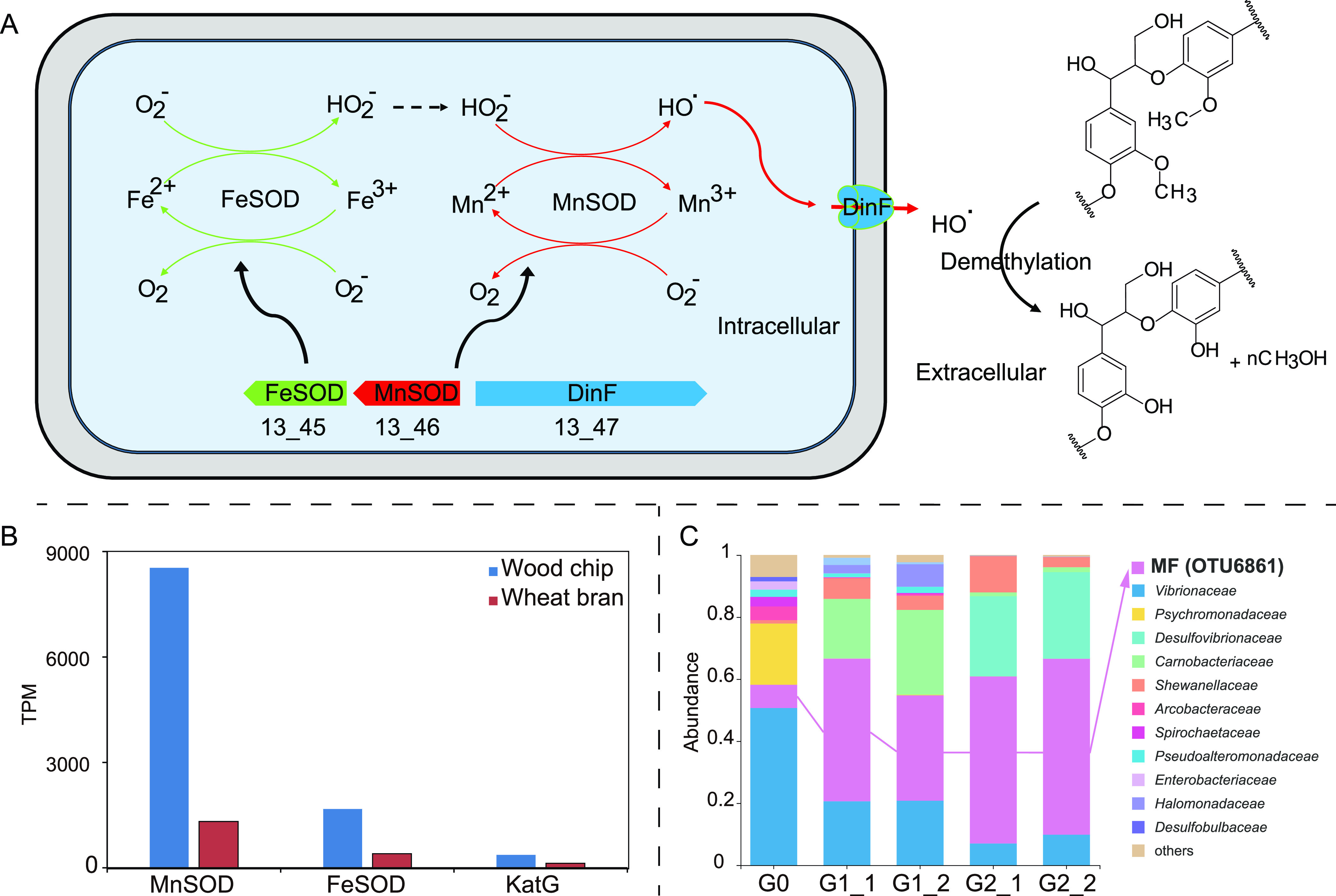
Proposed mechanism of lignin oxidation by MF bacteria and the bacterial community succession upon incubation with lignin. (A) Schematic diagram of the proposed mechanism of lignin oxidation by MF bacteria. We hypothesized that hydrogen peroxide produced by FeSOD is provided to MnSOD, and MnSOD then reduces hydrogen peroxide to hydroxyl radicals. The hydroxyl radicals produced in the cell are promptly excreted out of the cell by the DinF and oxidize the lignin by demethylation outside the cell. (B) Transcriptional abundance of the genes of MAG B6 involved in lignin oxidation in wood chips and wheat bran enrichments. MnSOD and FeSOD showed higher transcriptional activity in wood chip enrichment than in wheat bran enrichment. (C) Succession of bacterial community incubated with lignin as the sole organic carbon and energy source. Details are shown in the supplemental material (https://doi.org/10.5281/zenodo.7050448).

The genes upstream and downstream of the MnSOD gene in MAG B6 were annotated as encoding iron superoxide dismutase (FeSOD) and the efflux enzyme DinF, respectively (Fig. 5A). DinF belongs to the subfamily of multidrug and toxic compound extrusion (MATE)-like protein and is involved in the extrusion of reactive oxygen species (47). Since MnSOD from MAG B6 did not have signal peptides or encapsulin gene adjacent to it, like DyP-type peroxidase from Rhodococcus jostii RHA1 (48), it was probably not secreted extracellularly. Here, we constructed a possible model of lignin oxidation by MAG B6 (Fig. 5A). FeSOD in B6 provided hydrogen peroxide from superoxide to MnSOD; MnSOD produced hydroxyl radical via one-electron reduction of hydrogen peroxide; finally, hydroxyl radical was promptly excreted out of the cell by DinF and oxidized the lignin outside the cell (Fig. 5A).

In addition, the cluster including MnSOD, FeSOD, and DinF genes was also detected in other MF-2 bacteria, while it was not found in most MAGs belonging to MF-1, MF-3, or MF-4 (except MAG B9).

Metatranscriptomic analysis showed that the transcripts of the genes encoding MnSOD and FeSOD in the wood chip enrichment were 4 to 6 times more abundant than in the wheat bran enrichment (Fig. 5B), which implied that MAG B6 may oxidize lignin or its derivatives *in situ* in the deep sea. Furthermore, the community succession with lignin as the sole carbon and energy source in the laboratory showed that the relative abundance of OTU6861, which shared the same 16S rRNA gene sequence as MAG B6, increased significantly with incubation time (0, 0.5, and 1 year) (Fig. 5C; for details, see the supplemental material at https://doi.org/10.5281/zenodo.7050448), which further suggested that it may oxidize and utilize lignin to grow.

It is thought that in the wood fall ecosystem, the degradation of wood polysaccharides is carried out mainly by macrofaunal borers such as *Xylophaga* spp. (38, 49). Researchers believed that bacteria, including the MF clade, use only small organic compounds, such as sucrose, directly derived from wood or excretions from these macrofaunal borers *in situ* (28, 38). Therefore, the oxidation of lignin in the wood fall and the keystone bacteria involved in this process remain poorly understood. In this study, we gained insight into the ecological role of MF-2 bacteria in the degradation of polysaccharides and oxidation of lignin *in situ* in the deep sea. Furthermore, based on the above results, we believe that MF members present in water columns are possibly involved in the depolymerization of POM, since carbohydrates are known as one of the main components of POM in marine water columns (50) and the extracellular enzyme activity of glycoside hydrolases is abundantly detected in POM (13). Additionally, MF bacteria are present in oligotrophic sediment (34), which may be sustained by decomposing and utilizing buried rDOM derived from POM and DOM by the microbial carbon pump (3) and buried refractory humic acid materials, as well as refractory components (such as peptidoglycan) derived from the cell walls of dead microbes.

### Protein hydrolysis and utilization by subgroups MF-1, -3, and -4.

Bacteria of the MF-1, -3, and -4 subgroups dominated the communities of protein enrichments *in situ*, especially MF B9 and MF B8, both of which belong to subgroup MF-4 (Fig. 2B). From the B9 and B8 MAGs, 119 and 82 peptidase-encoding genes were retrieved, respectively (see Tables S9 to S11 at https://doi.org/10.5281/zenodo.7050448). Notably, approximately half of the peptidases encoded by MF B8 and B9 possess the N-terminal signal peptide (see Tables S10 and S11 at https://doi.org/10.5281/zenodo.7050448), indicating that they are secreted extracellularly and responsible for the degradation of extracellular protein and/or peptide (51). Moreover, some genes encoding aminotransferases and oxidative deaminases involved in amino acid metabolism were also detected, as well as central amino acid metabolism or degradation pathways, such as those for alanine, aspartate, and glutamate (Fig. 4B; also, see Table S12 at https://doi.org/10.5281/zenodo.7050448).

Furthermore, metatranscriptomic data sets from two enrichments with fish scales and fish tissue showed that MAGs B9 and B8 exhibited high transcriptional activities of functional genes responsible for polypeptide degradation, in addition to amino acid metabolism (Fig. 3A). Specifically, 78.8% and 48.3% of transcripts in the two consortia assigned to these secreted peptidases were generated from MAG B9 and B8, respectively, indicating that both bacteria contributed significantly to the degradation of polypeptides *in situ* in the deep sea.

### Differentiation among MF bacterial subgroups in nitrogen fixation and nitrogen source acquisition.

**FIG 6:**
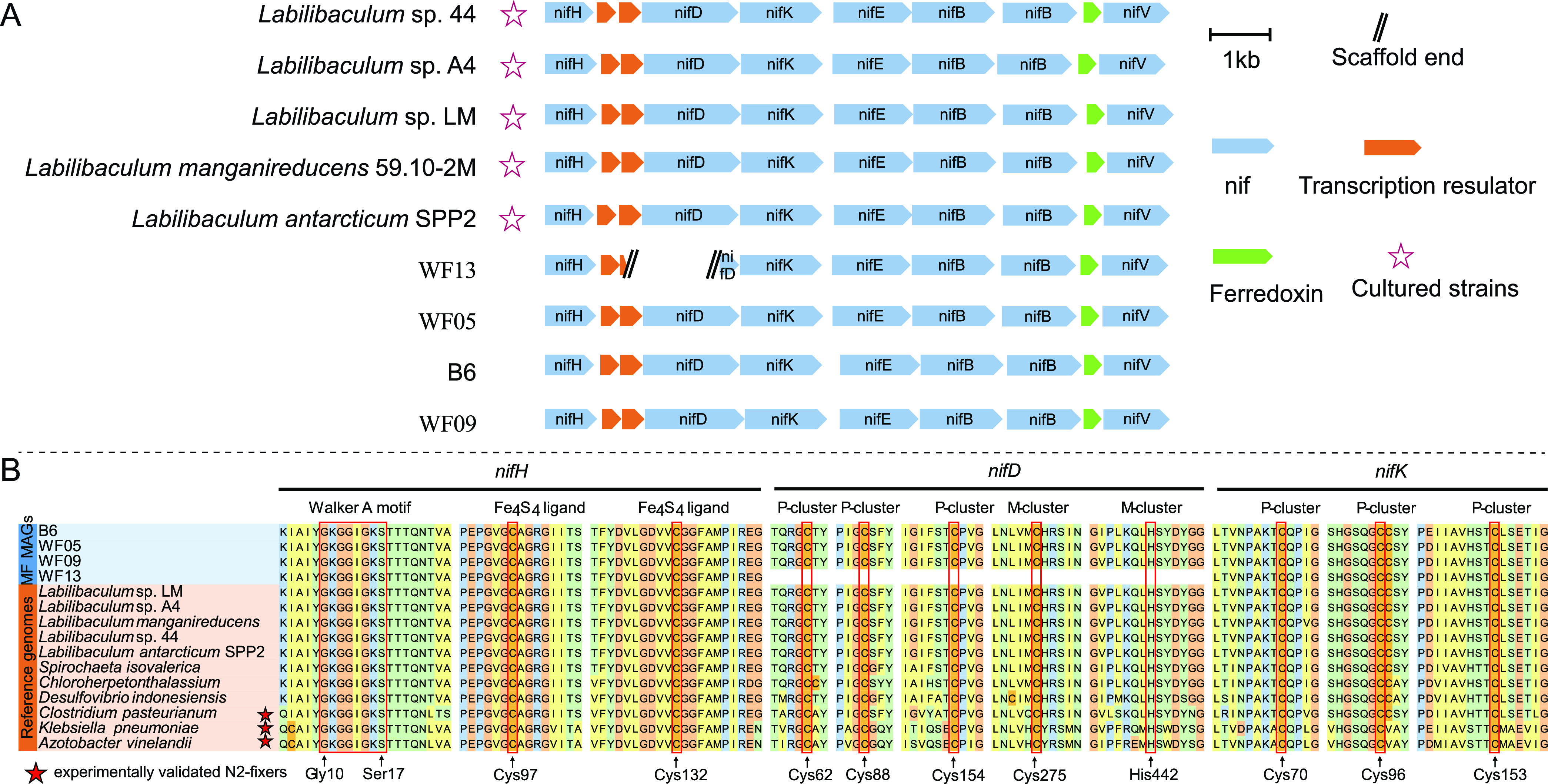
Genomic analyses of nitrogenase in MF-2 subgroup. (A) Schematic diagram of the nitrogenase gene cluster in the MF-2 MAGs from this study and reference strains. (B) Conserved amino acids for the key residues in three important proteins (*nifHDK*). *Labilibaculum* sp. strain 44, *Labilibaculum* sp. strain A4, *Labilibaculum* sp. strain LM, *L. manganireducens* 59.10-2M, and *Labilibaculum antarcticum* SPP2 are cultured strains; WF13, WF05, and WF09 were retrieved from wood falls (18, 38); and B6 is from our *in situ* enrichments in this study.

Nine of 10 members of subgroup MF-2 harbored a complete nitrogen fixation gene cluster (*nifHDKEBV*), along with related transcriptional regulatory genes (Fig. 6A). In contrast, the MF-1, MF-3, and MF-4 bacteria dominating the animal tissue enrichments did not encode the above-mentioned nitrogenase (Table 1; also, see Table S12 at https://doi.org/10.5281/zenodo.7050448). The NifHDK enzymes encoded by MF-2 bacteria contain important conserved residues (52), as in other nitrogen-fixing bacteria (Fig. 6B). These NifHs share relatively high amino acid similarities (67 to 72%) with those of nitrogen-fixing bacteria Clostridium pasteurianum, Azotobacter vinelandii, and Klebsiella pneumoniae (53) (see Table S13 at https://doi.org/10.5281/zenodo.7050448). Moreover, we observed nitrogen fixation in the laboratory by an MF-2 isolate (*Labilibaculum* sp. strain LM in Fig. 2A) of mangrove sediment origin (data not shown). These results indicated that MF-2 members may be nitrogen-fixing bacteria.

In addition to nitrogen fixation genes, a gene cluster for assimilatory nitrate reduction (*narB*, *nirBD*, and *nrtABCD*) was detected only in MAG B6, not in the genomes of MF-1, -3, and -4 bacteria (see Table S12 at https://doi.org/10.5281/zenodo.7050448). The metatranscriptomic data revealed that these genes from MAG B6 involved in nitrogen fixation and assimilatory nitrate reduction were transcribed during polysaccharide/lignin degradation, although their transcript levels were low (see Table S12 at https://doi.org/10.5281/zenodo.7050448). These results indicated that the pattern of nitrogen source acquisition may be one of the key metabolic features determining MF bacterial specialization and adaptation to different types of OM and that MF-2 members may play an important role in balancing the disproportion of nitrogen and carbon sources, especially when the available nitrogen sources are scarce and polysaccharide or lignin is used as a growth substrate.

This is the first report that *Marinifilaceae* bacteria within the phylum *Bacteroidetes* have nitrogen-fixing potential. A previous report showed that abundant nitrogen-fixing genes are derived from *Bacteroidetes* in the termite gut (54, 55), suggesting that *Bacteroidetes* may play a significant role in the nitrogen balance of the intestinal flora. Similarity, MF members are also detected in the gut of marine animals in addition to the surfaces of marine plants and animals, e.g., barnacles and corals (Fig. 1A and B) (56–58). Those MF bacteria may act as probiotics to provide ammonia nitrogen to the host by nitrogen fixation *in situ*. The wide distribution of MF-2 in the ocean (Fig. 1B) indicates the universality of nitrogen fixation in the marine environment, as was recently reported regarding the diversity of diazotrophs in deep-sea sediment (59).

### Versatile anaerobic respiration mechanisms of MF bacteria.

**FIG 7:**
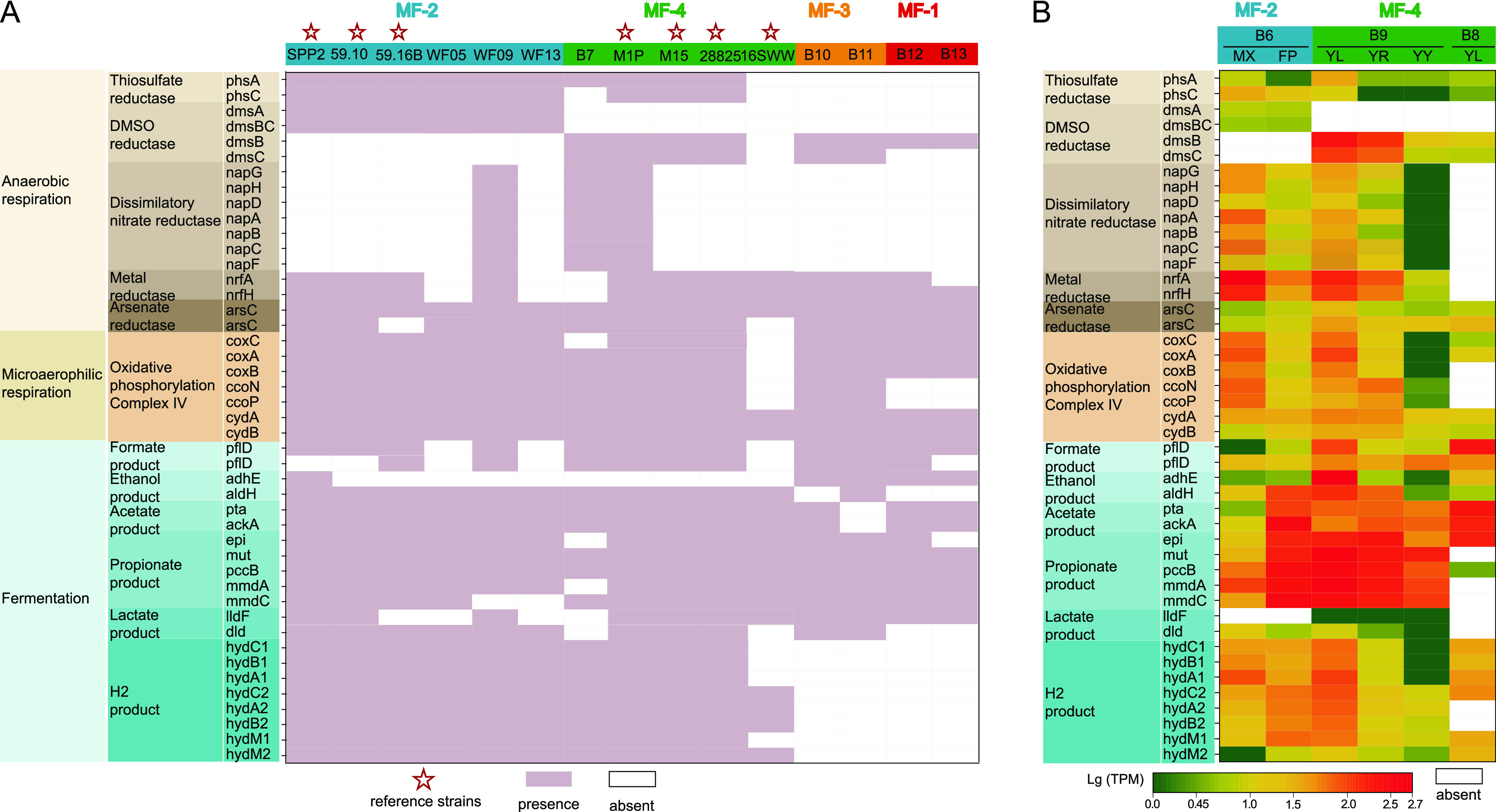
Cell respiration and fermentation pattern in MF members. (A) Genes present or absent in the genomes of MF members involved in cell respiration and fermentation. (B) Transcriptomic profiles of related genes in MAG B6, B8, and B9. SPP2, *Labilibaculum antarcticum* SPP2; 59.10, *Labilibaculum manganireducens* 59.10-2M; 59.16B, *Labilibaculum filiform* 59.16B; M1P, *Ancylomarina* sp. strain M1P; M15, Ancylomarina salipaludis SHSM-M15; 28825, Ancylomarina subtilis DSM 28825; 16SWW, Ancylomarina psychrotolerans 16SWW S1-10-2. B6 to B13 were obtained from our enrichments in this study; WF05, WF09, and WF13 were from public wood falls.

In addition to the microaerophilic respiration (*coxABC*, *ccoNP*, and *cydAB*), most MF bacteria possessed the potential to use thiosulfate, dimethyl sulfoxide (DMSO), and arsenate as the terminal electron acceptors but were incapable of sulfate reduction (Fig. 7A and B; also, see Table S14 at https://doi.org/10.5281/zenodo.7050448). In addition, both *L*. *manganireducens* 59.10-2M^T^ and Labilibaculum filiforme 59.16B^T^ have been confirmed to reduce Fe^3+^ and Mn^4+^ ions and/or oxides during glucose fermentation, presumably through a cytochrome *c* gene (34). This gene was also detected in most MAGs (Fig. 7A and B; also, see Table S14 at https://doi.org/10.5281/zenodo.7050448). At present, the metal-reducing (Fe^3+^ and Mn^4+^) bacteria mainly belong to *Firmicutes*, *Proteobacteria*, *Deferribacteres*, and *Deinococcus*-*Thermus* (60, 61). Moreover, MAG B6 and WF09 additionally possess a gene cluster for dissimilatory nitrate reduction (*napABCDFGH*) (Fig. 4 and 7A and B).

Fermentation was additionally found to be a common strategy among MF members, even in the presence of some alternative terminal electron acceptors. Most MF bacteria (B6-B13, WF05, WF09, and WF13) in this study had the potential to generate formate (*pflD*), ethanol (*aldH* and *adhE*), acetate (*acdAB* and *ackA*), propionate (*mmdAC* and *pccB*), and lactate (*lldF* and *dlD*) via fermentation (Fig. 7A and B; also, see Table S14 at https://doi.org/10.5281/zenodo.7050448). In addition, almost all members of MF-2 and MF-4 possess three groups of [FeFe]-hydrogenases (groups A3, B, and C1; *hydABC*, *hydS*, and *hydM*) (Fig. 7A and B; also, see Fig. S8A and B and Table S14 at https://doi.org/10.5281/zenodo.7050448), all of which are typically involved in strictly anaerobic fermentation for hydrogen production (62). These fermentation products can serve as carbon sources for SRB, e.g., *Desulfobulbaceae* and *Desulfobacteraceae*, which are and/ or energy sources also dominant in animal tissue and plant detritus enrichments (32).

Metatranscriptomics analysis showed that all the genes involved in anaerobic respiration and fermentation were actively transcribed in MAG B6, MAG B8, and MAG B9 during corresponding OM degradation (Fig. 7B; also, see Table S14 at https://doi.org/10.5281/zenodo.7050448). In particular, the genes encoding putative Fe^3+^ and Mn^4+^ ion and/or oxide reductases exhibited obviously high transcriptional levels regardless of the organic substrates, and the genes encoding propionate fermentation (*mmdAC* and *pccB*) showed significantly high expression levels (Fig. 7B), which suggested that MF members may prefer Fe^3+^ and Mn^4+^ ions and/or oxides as electron acceptors and ferment OM to generate propionate during OM degradation *in situ* in the deep sea. The energy gained from Fe^3+^ and/or Mn^4+^ reduction is high, comparable to that from using oxygen as the electron acceptor. The above results indicated that MF use various substances as terminal electron acceptors for anaerobic respiration coupled with diverse fermentation types during the degradation of various OM *in situ* in the deep sea.

Biodegradation of OM inevitably results in oxygen depletion, which not only occurred in the past but also continues to occur globally in the oceans, e.g., in oxygen minimum zones (63) and wood falls (38). Similarly, the globally increasing anoxic areas are the consequence of abundant OM input from rivers and eutrophication in estuarine areas (64). Moreover, fermentation is often found in other OM-rich environments, such as cold seeps (65) and petroleum seeps in deep-sea sediments (66).

### Conclusion.

**FIG 8:**
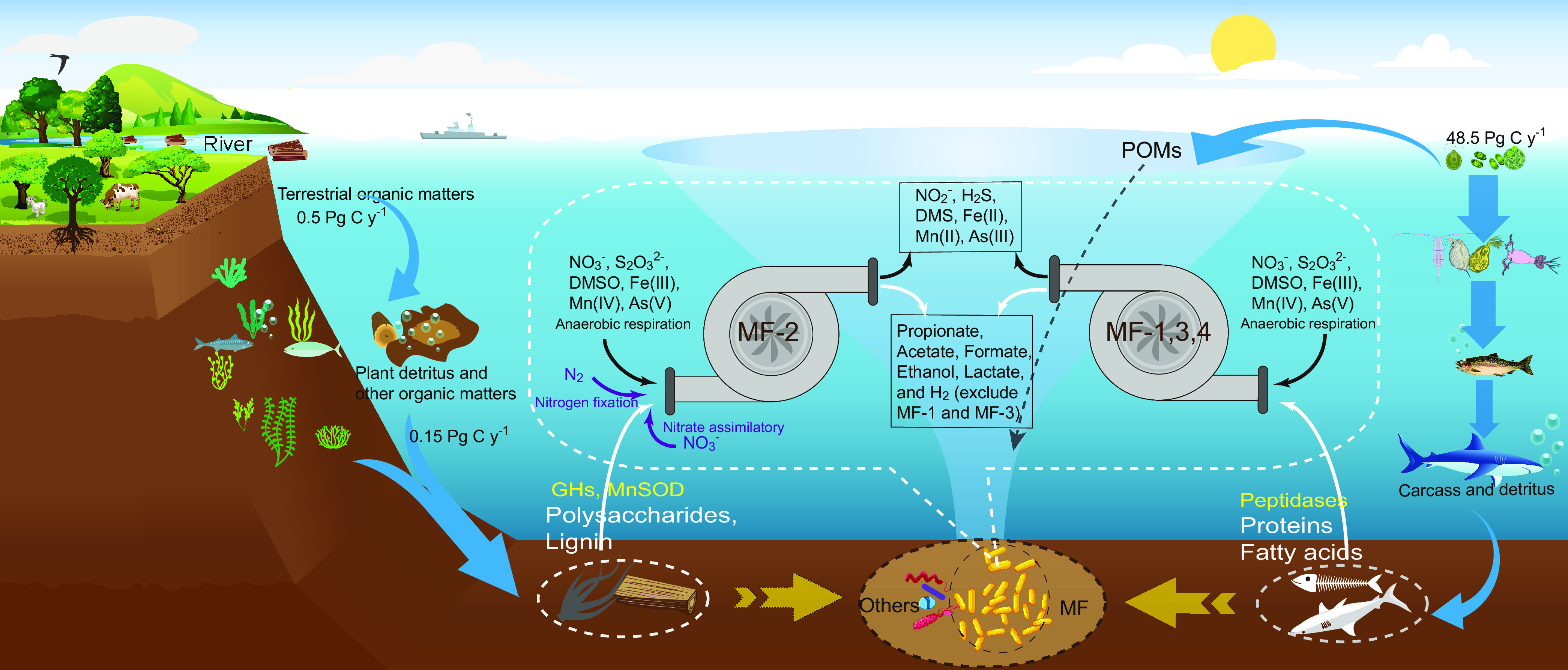
Schematic depiction of the ecological role of MF in the global oceans. MF members are the predominant group in the assemblages thriving on newly input plant detritus and animal tissue in the deep sea. Their wide distribution in marine environments and metabolic potentials in OM mineralization highlight their role in ocean carbon cycling. Within this group, bacteria diverge not only in phylogeny but also in metabolisms for the degradation of protein, polysaccharides, and lignin, but they share methods of energy conservation via organic fermentation and anaerobic respiration.

This study highlights the ecological roles of the bacteria of the family *Marinifilaceae* within the phylum *Bacteroidetes* in the depolymerization, degradation, and transformation of POM derived from animal and plant detritus *in situ* in the deep sea under anoxic conditions, simultaneously coupled with nitrogen, sulfur, and metal elemental biogeochemical cycling, as summarized in the diagram in Fig. 8. Bacteria of subgroup MF-2 thriving in plant detritus sunk into the deep sea (such as wood fall), possess overwhelming advantages in polysaccharide hydrolysis, lignin oxidation, and nitrogen source acquisition (nitrogen fixation) that facilitates their survival during a deficit of nitrogen sources inside the plant detritus. In contrast, MF-1, MF-3, and MF-4 are the predominant members of the microbial assemblages thriving on animal tissues and remains (such as whale fall) and specializing in hydrolyzing and utilizing proteins or polypeptides. In addition to OM mineralization, MF members can conduct fermentation and diverse anaerobic respiration. Overall, MF members are key players in OM mineralization and are coupled with sulfur, nitrogen, and metal elemental biogeochemical cycles *in situ* in the deep sea.

## MATERIALS AND METHODS

### Incubation samples.

Previously, we obtained a large number of plant detritus and animal incubation samples from the deep-sea region of the marginal sea (the South China Sea) and ocean (the Pacific Ocean and the Indian Ocean) using deep-sea *in situ* microbial incubators (32). In this study, we selected five representative incubation samples to further analyze the metabolic potential of MF subgroup in the *in situ* environment by analyzing their metagenomic and metatranscriptomic data. The five incubations, emended with wood chips, wheat bran, fish scales, fish tissue, and fish oil (DHA and EPA), were incubated at a flat-topped seamount in the Pacific Ocean (20.4059567° N, 160.7700883° E; 1,622 m water depth) for 348 days. The detailed enrichment process and organic substrates are described in the previous article (32).

### DNA and RNA extraction.

The enriched biomass in the liquid phase was filtered on a 0.22-μm-pore-size polycarbonate membrane (Millipore, USA); one half was used for DNA extraction and the other half for RNA extraction. Total DNA was extracted and purified with a DNeasy PowerWater kit (Qiagen, Germany) according to the manufacturer’s protocol. Total RNA was extracted using the RNeasy minikit (Qiagen, Germany) according to the manufacturer’s instructions. The concentration and quality of the extracted DNA and RNA were determined using a NanoDrop2000 spectrophotometer (Thermo Scientific) and gel electrophoresis, respectively. All DNA and RNA samples were stored at −80°C until further processing.

### Cloning of 16S rRNA gene and sequencing.

The extracted DNA was amplified using the broad-range bacterium-specific 16S rRNA gene primer pair 27F (5′-AGAGTTTGATCCTGGCTCAG-3′) and 1492R (5′-ACGGCTACCTTGTTACGACT-3′). Amplicons were purified by using the QIAquick PCR purification kit (Qiagen Inc., Valencia, CA), and clone libraries were constructed with the TOPO TA cloning kit (Invitrogen) according to the manufacturer’s instructions. Inserts from the clones were amplified with the primers M13F and M13R and Sanger sequenced from both ends by the Majorbio Company (Shanghai, China).

### Global distribution pattern.

To reveal the global distribution of the enriched MF species in the deep-sea enrichments, we collected microbial data containing MF reads by using the IMNGS server (https://www.imngs.org; last accessed on 5 May 2019), which pigeonholes all available raw sequence read archives retrieved from the International Nucleotide Sequence Database (GenBank, DDBJ, and EMBL) and allows users to conduct comprehensive searches of small-subunit (SSU) rRNA gene sequences. In this study, representative sequences (the nearly full-length 16S rRNA gene) were subjected to a similarity search against the IMNGS database using a similarity threshold of 95% and a minimum length of 200 bp. A total of 1,026 samples were retrieved, and their metadata, including source, latitude, longitude, and depth of seawater, were obtained from NCBI (see Table S1 at https://doi.org/10.5281/zenodo.7050448).

### Metagenomic sequencing, assembly, and binning.

Metagenomic library preparation and DNA sequencing using Illumina NovaSeq 6000 platform (paired-end (PE) 150-bp mode) were conducted at the Majorbio Company (Shanghai, China). Raw metagenomic reads were subjected to quality control (QC) processing by using the fastp v0.19.3 with default parameters (67). Possible animal or plant reads from substrates were removed by mapping to the genomes of *Pinus*, Triticum aestivum, and Larimichthys crocea using Bowtie2 (identity ≥ 0.9) (68). Filtered reads were individually assembled *de novo* by MetaSPAdes v3.13.0 with the settings “-k 21,33,55” (69). Previous studies on wood falls showed the presence of MF members (18, 38), so we additionally retrieved metagenomic data from the MG-RAST database (https://www.mg-rast.org/). Their accession numbers are mgs476148, mgs476151, mgs476154, mgm4623131.3, mgm4623132.3, and mgm4623133.3. These data were processed for QC and coassembled using the same method.

The binning process was performed by using the Metawrap pipeline v1.2.1 based on three methods, metabat2, maxbin2, and concoct (70). Two modules, bin_refinement and reassemble_bins, in the Metawrap pipeline were then implemented with the parameters -c 50, -x 10 (70). Afterward, MAGs with >50% completeness and <10% contamination were picked for subsequent analysis. All binning results were combined and dereplicated using dRep (-comp 40 -con 15 –run_tax -g options). After dereplication, a total of 110 dereplicated MAGs were obtained from our five deep-sea *in situ* incubations and 35 MAGs were obtained from wood falls. The completeness and contamination of each MAG were estimated by CheckM v1.0.12 (71). The coverage of each MAG in each sample was calculated by using Salmon software in the Metawrap pipeline with the quant_bins module (70). The relative DNA abundance (from the metagenome) of each MAG in each sample was calculated as its coverage divided by the total coverage of all MAGs in each metagenomics data set.

### Taxonomic assignments and ANI calculation.

Taxonomic assessment of each MAG was performed using GTDB-Tk with the database of GTDB R04-RS89 (72). A total of 11 distinct MAGs belonged to MF and were used for the subsequent analysis. The ANI values between these MAGs and cultured MF bacteria were calculated with FastANI v1.1 (73).

### Functional annotations.

Protein-coding genes of each MAG were predicted using Prodigal v2.6.3 (74). Protein sequences were functionally annotated against databases, including KEGG (75), eggNOG (76), Pfam (77), MEROPS (78), and CAZy (79). The online software KAAS v2.1 (75) (https://www.genome.jp/kegg/kaas/) was applied for homology searching against the KEGG database with the GHOSTZ program. eggNOG-mapper v1.0.3 software was used to annotate with the Diamond BLASTP (v0.8.36.98) method (76). To identify carbohydrate degradation-related enzymes, we used the online dbCAN2 metaserver (http://bcb.unl.edu/dbCAN2/) by HMMER against the CAZy database (79). Pfam 31.0 (77) was used as the reference database for the annotation of peptidases, aminotransferases, and transporters of oligopeptides and amino acids by HMMER v3.1b2 (cutoffs: e value, 1e−10; best hits reserved). Additionally, the MEROPS database (release 12.1) (78) was used for peptidase annotation by Diamond BLASTP v0.8.36.98 (cutoff: e value, 1e−10; best hits reserved) (80). When a protein sequence was annotated to the same peptidase family with the above two databases, its annotation result was accepted and then used for subsequent analyses. The prediction of signal peptides was carried out by using the online SignalP-5.0 server (https://services.healthtech.dtu.dk/service.php?SignalP-5.0). The three-dimensional structures of certain key encoded proteins were predicted by using the online Phyre2 web portal (81).

### Metatranscriptomic sequencing and mapping.

Metatranscriptomic library preparation and cDNA sequencing using Illumina NovaSeq 6000 platform (PE 150-bp mode) were conducted at the MAGIGENE Company (Guangzhou, China). Raw metatranscriptomic reads were processed for QC by using fastp v0.19.3 with default parameters (67), and the resulting clean reads without rRNA were mapped to each MAG using Bowtie2 with default parameters (68). Afterward, the counting of fragments (PE reads) assigned to each gene in the MAG was carried out using the FeatureCounts program with the parameters -p -F GTF -g ID -t CDS -s 1 -M –fraction (82). The transcript of each gene in each MAG was normalized to the number of transcripts per million (TPM) (83). The relative cDNA abundance (from the metatranscriptome) of each MAG was calculated as its transcript number divided by the sum of all MAG transcripts in each sample.

### Phylogenetic analysis.

16S rRNA gene homology searches within GenBank were performed using BLASTn. A total of 338 sequences belonging to MF were retrieved (see Table S2 at https://doi.org/10.5281/zenodo.7050448). Multiple-sequence alignment of the 16S rRNA gene amplicon was performed using the MAFFT program with default parameters, and a phylogenetic tree was constructed by the neighbor-joining method (https://mafft.cbrc.jp/alignment/server/). For the phylogenetic tree of hydrogenase, a neighbor-joining tree was constructed by using MEGA v7.0 software (84). The reference hydrogenase catalytic subunits were retrieved from a previous study (62). For draft MAGs, the phylogenomic tree was built by the up-to-date bacterial core gene (UBCG) method (85). The reference genomes were retrieved from the EzBioCloud database (https://www.ezbiocloud.net/) in May 2021.

### Data availability.

The raw data including metagenomic sequences, metatranscriptome sequences, and high-throughput sequencing of 16S rRNA gene sequences, and MAGs have been deposited in NODE (https://www.biosino.org/node/) with the accession numbers OEX011321, OEX011323, OEX011325, and OEZ007098, respectively. The nearly full-length 16S rRNA sequences belonging to MF acquired in this study have been deposited in GenBank under the accession numbers ON228496 to ON228518. The supplemental material is available at https://doi.org/10.5281/zenodo.7050448.
